# Rougeur oculaire: penser à la rosacée

**DOI:** 10.11604/pamj.2015.21.77.4097

**Published:** 2015-05-29

**Authors:** Othman Charhi, Rajae Daoudi

**Affiliations:** 1 Mohammed V University Souissi, Faculty of Medicine, Department “A” of Ophthalmology, Rabat, Morocco

**Keywords:** Rougeur oculaire, rosacée, baisse de l′acuité visuelle, redness, rosacea, decreased visual acuity

## Image en medicine

Patiente est âgée de 50 ans sans antécédents particulier qui consulte pour rougeur oculaire + baisse de l'acuité visuelle inaugurale sans douleur oculaire. L'examen retrouve en OD une meïbomite, une rougeur oculaire conjonctivale, une opacité cornéenne proche du limbe avec un appel vasculaire cornéen, une infiltration limbique collectée, une kératite ponctuée superficielle. L'examen cutané du visage retrouve une légère éruption vésiculo-pustuleuse (flèche noir) prenant la partie supérieure de l'hémiface droite et le nez. Trois diagnostiques on été évoqué: rosacée, zona et abcès cornéen. La patiente a été mise sous Cyclines oral associé à l'azythromycine, relayé par une corticothérapie. Devant la réponse positive au traitement, la diagnostic de rosacée oculaire a été retenue. La rosacée est une infection chronique d’étiologie mal connu qui peut prêter à tort avec des infections bactérienne ou virale Les signes cliniques évocateurs sont généralement une meibomite avec télangiectasies du bord libre de la paupière, une blépharite, des chalazions à répétitions, une rougeur oculaire avec des lésions cornéennes inflammatoires proche du limbe (flèche blanche) …La présence d'un appel vasculaire cornéen (flèche jaune) témoigne de l’évolutivité de la maladie Le traitement associe un lavage au sérum physiologique et des larmes artificielles, des cyclines orales et de l'azytromicine topique. Le traitement corticoïde est efficaces pour réduire l'inflammation et les appels vasculaires.

**Figure 1:**
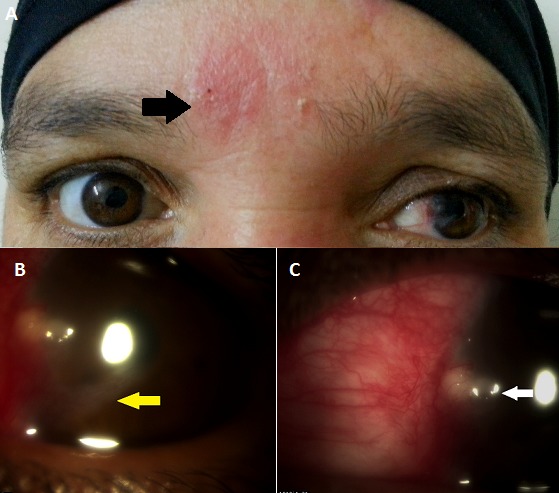
A) examen du visage retrouvant les lésions vésiculopustuleuse (flèche noir); B) appel vasculaire cornéen opacifiant la cornée; C) inflitrat inflammatoire proche du limbe (flèche blanche)

